# Evaluating the Potential of Microdosing 1cp‐LSD for the Treatment of Canine Anxiety: A One‐Month Case Study

**DOI:** 10.1002/vms3.70486

**Published:** 2025-07-10

**Authors:** Elisa Hernández‐Álvarez, Lucas F. Borkel, Jaime Rojas‐Hernández, Domingo J. Quintana‐Hernández, Ignacio García‐Serrano, Tobías Fernández‐Borkel, Manuel Zumbado, Luis Alberto Henríquez‐Hernández

**Affiliations:** ^1^ Faculty of Veterinary Universidad de Las Palmas de Gran Canaria Arucas Spain; ^2^ Asociación Científica Psicodélica, Canary Islands Las Palmas de Gran Canaria Spain; ^3^ Health Sciences Faculty Universidad de Las Palmas de Gran Canaria Las Palmas de Gran Canaria Spain; ^4^ Asociación Canaria para el Desarrollo de la Salud a través de la Atención Las Palmas de Gran Canaria Spain; ^5^ Faculty of Psychology Universidad del Atlántico Medio Las Palmas de Gran Canaria Spain; ^6^ Human Dog, Canary Islands Las Palmas de Gran Canaria Spain; ^7^ Department of Forensic and Neurodevelopmental Sciences Institute of Psychiatry Psychology & Neuroscience, Kings College London UK; ^8^ Unit of Toxicology Clinical Science Department Universidad de Las Palmas de Gran Canaria Las Palmas de Gran Canaria Spain

**Keywords:** 1cp‐LSD, anxiety, animal behaviour, animal consciousness, dogs, LSD, microdosing, Psychedelics

## Abstract

This pilot study explored the potential of microdosing 1‐cyclopropionyl‐d‐lysergic acid diethylamide (1cp‐LSD) to treat canine anxiety. A single‐case study was conducted on a 13‐year‐old female dog with severe separation anxiety, who was treated with 5 µg of 1cp‐LSD every 3 days for a month. Anxiety was assessed before, after, and 1 month following treatment using a validated questionnaire. The owner's attachment style was assessed using a validated scale. The dog's anxiety score significantly decreased from 29 (severe) to 14 (moderate) after treatment. A reduction in anxiety levels was observed, characterized by decreased destructive behaviour and shorter durations of vocalization. This improvement was sustained 1 month following treatment, although vocalization frequency increased. These findings suggest potential therapeutic efficacy of microdosing 1cp‐LSD in managing canine anxiety; however, the absence of a control group makes it difficult to determine whether the observed effects are due to 1cp‐LSD, owner bias, or natural variability in the dog's behaviour. Additional studies with blinded protocols and larger sample sizes are necessary to validate these findings and further explore the impact of owner attachment on canine anxiety.

## Graphical abstract

A 13‐year‐old dog with severe separation anxiety received 5 µg of 1cp‐LSD every 3 days for 30 days. Anxiety scores decreased from 29 (severe) to 14 (moderate) at the end of treatment, and scored 15.5 1 month later. These findings suggest a potential anxiolytic effect of microdosing 1cp‐LSD, though further controlled studies are needed.

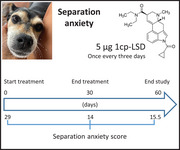

## 1 Introduction

Anxiety is one of the main issues affecting the health of dogs (*Canis lupus familiaris*), with an estimated prevalence of 14–20% (Salonen et al. 2020). Currently, there are approximately 9.3 million registered dogs in Spain, according to official data for the year 2022 (Statista 2023), which means that 1.3–1.8 million of them suffer from this problem. Although there are various types of anxiety, one of the most prevalent is separation anxiety (Salonen et al. 2020, Sherman and Mills 2008). Anxiety in dogs causes significant distress for both the animal and its caregivers. Among its symptoms are hyperactivity, attention deficits, aggression towards strangers, vocalization, continuous barking, and compulsive behaviours like tail‐chasing or inappropriate urination/defecation (Salonen et al. 2020, Flannigan and Dodman 2001). These behaviours can become unbearable for owners, who sometimes decide to rehome affected animals through adoption or abandonment. While statistics on the reasons for adoptions or abandonments are lacking, given the high incidence of abandonment in our country and the prevalence of anxiety (Crespo‐Garay 2021), the correlation is plausible. The aetiology of canine anxiety disorders is complex. It involves not only factors related to the animal's development, socialization, and environment (Hernández‐Garzón 2012, Hernández‐Garzón 2012) but also intrinsic aspects of physical health. In this regard, a recent study has established an association between *Toxoplasma gondii* infection and anxiety disorders in dogs, suggesting that neurobiological changes induced by the parasite may contribute to anxiety‐like behaviours (Dini et al. 2025). Various predisposing and triggering factors contribute to anxiety (Salonen et al. 2020), with environmental factors playing a decisive role in the onset of canine anxiety (Salonen et al. 2020, Overall et al. 2014). The treatment of separation anxiety is complex and prolonged, requiring owner education, environmental adjustments, and behavioural therapy (Hernández‐Garzón 2012). The existing literature shows an association between the anxiety and depressive processes of the owners and their pets (Xestal 2022). The owner–dog bond plays a crucial role, as dogs perceive and respond to the emotional environment created by their caregivers (Sundman et al. 2019). Studies indicate that owners’ personality traits correlate with those of their dogs, likely due to shared environments, emotional contagion, or selective matching (Gobbo and Zupan 2020, Turcsán et al. 2012). Moreover, the percentage of owners who suffer from separation anxiety is double that of dogs experiencing it when they are apart, with women tending to anthropomorphize their pets more (Xestal 2022). Different scientific studies have shown this association, indicating that the mental health and character of the owners condition the mental health and character of their dogs (Pereira et al. 1987, 2021).

The relationship between serotonin and anxiety disorders has been known for decades (Baldwin and Rudge 1995). Although the pathophysiology of anxiety is multifaceted and encompasses multiple neurotransmitter systems and neuronal receptors, anxiety in humans and dogs involves serotonin receptor dysfunction (Akimova et al. 2009, Vermeire et al. 2009). Despite these similarities, selective serotonin reuptake inhibitors (SSRIs) are effectively used for human anxiety (Zangrossi et al. 2020) but are not considered as first‐line therapy for the treatment of canine anxiety. Novel non‐pharmacological treatment protocols like faecal microbiota transplantation (Chinna Meyyappan et al. 2020), which is now implemented in human medicine, have not been tested for the treatment of canine anxiety. The treatment of canine anxiety includes behavioural, environmental, and pharmacological interventions (Arguelles et al. 2024). However, like in humans, these treatments often fail (e.g., 65.1% vs. 51.3% of improvement among SSRI and placebo groups, respectively), require long‐term use, and can have adverse effects (e.g., serotoninergic syndrome with SSRIs and rebound anxiety with benzodiazepines) (Dantas and Ogata 2024). In this regard, clinical veterinarians manage canine anxiety without established treatment protocols, often relying on limited scientific evidence and lacking sufficient tools to control this condition (ESVCE 2022). Cannabinoid derivatives (CBD) are among these, but their effectiveness for anxiety control in dogs is currently very limited (Corsato Alvarenga et al. 2023). Therefore, pharmacological alternatives are needed.

For decades, psychedelic substances have shown considerable therapeutic potential (Lowe et al. 2022), mainly through the activation of serotonin receptors (Kwan et al. 2022). Although banned in the late 1950s and classified as Schedule I drugs, later research has shown these substances to be safe (Henriquez‐Hernandez et al. 2023), leading to a renewed interest within the scientific community. The emergence of legal analogues has allowed researchers to propose new studies in this field of knowledge (Brandt et al. 2020).

The physio pathological similarities between canine and human anxiety, coupled with the promising results of psychedelic use in humans for the treatment of this condition, have led us to propose a study whose aim was to assess the effects of microdoses of 1‐cyclopropionyl‐d‐lysergic acid diethylamide (1cp‐LSD), administered for 1 month, as a treatment for anxiety in one dog. The selection of 1cp‐LSD as a potential treatment for canine anxiety is based on its pharmacological properties compared with existing therapeutic options. Unlike SSRIs, which primarily increase extracellular serotonin levels by blocking its reuptake, serotonergic psychedelics such as LSD and its derivatives act as direct agonists at multiple serotonin receptor subtypes, particularly 5‐HT2A (Wacker et al. 2017). This receptor plays a key role in emotional regulation, which may contribute to a more rapid anxiolytic effect compared with the delayed onset of SSRIs (Brouwer and Carhart‐Harris 2021). Additionally, SSRIs have been associated with emotional blunting and adverse effects such as serotonin syndrome and withdrawal symptoms, making their long‐term use in canine patients challenging (Dantas and Ogata 2024). Cannabidiol (CBD) has also been proposed as an alternative treatment; however, its anxiolytic efficacy in dogs remains poorly established, with inconsistent findings across studies (Yu and Rupasinghe 2021). In contrast, emerging evidence suggests that psychedelic compounds at low doses may modulate mood and anxiety‐related behaviours with minimal psychoactive effects (Cameron et al. 2020), warranting further investigation into their potential as novel therapeutics for veterinary applications (Henriquez‐Hernandez et al. 2024).

In a previous study involving a single experimental administration, we observed that the substance was safe and exerted anxiolytic effects (Henriquez‐Hernandez et al. 2024). The present study reports the results of this 1‐month pilot trial and represents a necessary preliminary step before conducting a clinical trial.

## 2 Materials and Methods

### 2.1 Patient

The patient enrolled in the trial was a 13‐year‐old female dog, weighing 13 kg (70 cm length), of mixed breed and spayed. The owner was a 33‐year‐old middle‐class female with a college degree who had had a lifelong companionship with dogs, starting from her teenage years. The dog has always been part of the family, but has been living alone with the owner for the past year. The animal resides in a single‐story house with access to a garden and open spaces, alongside another dog. Initial patient eligibility was assessed by a veterinary professional specializing in canine ethology. Prior to the present study, a microdose of 1cp‐LSD was administered to the same patient in a single dose to observe the effects of the drug in the canine species, as no literature existed on this matter. The results of this single‐dose pilot trial demonstrated that microdosed 1cp‐LSD was safe and effectively reduced anxiety in the animal (Henriquez‐Hernandez et al. 2024). The entire treatment, lasting 1 month, was conducted in‐house without altering the patient's usual environment. The study was approved by the Animal Experimentation Ethics Committee of the University of Las Palmas de Gran Canaria (ref no. OEBA_ULPGC 02/2024).

### 2.2 Instruments for Measuring Canine Anxiety and Human Attachment

According to the initial assessment by the veterinary ethologist, the patient exhibited signs of separation anxiety. To objectively assess the patient's anxiety level, a validated and previously published scale was utilized (Parthasarathy and Crowell‐Davis 2006). This instrument, specifically designed to objectively evaluate canine separation anxiety, consists of 17 questions, rated on a scale from 0 to 21 points or more. Scores range as follows: 0 to 3 points indicate no separation anxiety; 4 to 8 points suggest mild attachment disorder; 9 to 15 points indicate moderate separation anxiety; 16 to 20 points indicate marked separation anxiety; and 21 points or more indicate severe separation anxiety. A total of three anxiety evaluations were conducted on the patient: the first, 1 day before starting the treatment; the second, 1 day after completing the treatment; and the third, 1 month after completing the treatment. The Spanish version of the questionnaire is available as supplementary material (Table S1). The numbers in parentheses indicate the scoring of each item within each question, with the total score reflecting the intensity level of canine separation anxiety.

We utilized a non‐romantic attachment scale, completed by the dog's owner, which is a validated questionnaire comprising 11 questions aimed at assessing the degree of attachment not related to romantic partners, distinguishing between fearful, avoidant, secure, and anxious attachment (Casullo and Fernández‐Liporace 2005). The attachment assessment was conducted the day before the treatment commenced. Evaluating the owner's attachment style is essential for understanding the patient's behaviour within their home environment, considering the established link between human and canine anxiety (Pereira et al. 1987, 2021).

### 2.3 Dosage of 1cp‐LSD and Treatment Protocol

1cP‐LSD is a semi‐synthetic LSD analogue that has been available for research since July 2019 (Brandt et al. 2020). It interacts with adrenergic and dopamine receptors and acts as a serotonin 5‐HT receptor agonist (Nichols 2016). The substance hydrolyzes to LSD via blood carboxylesterases, acting as a prodrug (Brandt et al. 2020).

A total of 5 µg, equivalent to 0.38 µg/kg of body weight, was administered orally to the subject every 3 days (Fadiman 2011). Since no pharmacokinetic studies of LSD have been conducted in dogs, the selected dose was extrapolated from human data, acknowledging potential interspecies differences in substance metabolism and effect. To ensure an appropriate dosage, the patient's body surface area was calculated using the Du Bois formula (Du Bois and Du Bois 1989) and normalized to a standard human of 80 kg and 180 cm, as follows (Equation 1):

(1) $$\mathrm{BSA}\;\left(m^{2}\right)=0.007184\times \mathrm{BW}{\left(\mathrm{kg}\right)}^{0.425}\times L{\left(\mathrm{cm}\right)}^{0.725},$$

where BSA is defined as body surface area, BW as body weight, and L as length (nose‐to‐base of the tail). A ratio is then established between the body surface area of a human weighing 80 kg and measuring 180 cm in height, set at 1.99 m^2^, and the body surface area of the dog. Finally, this ratio is multiplied by the administered dose. The resulting estimated dose was 21.4 µg, close to the typical microdosing range employed in humans (5–25 µg) (Liechti and Holze 2022). This dosage has been demonstrated to be safe in human medicine (Liechti and Holze 2022, Borkel et al. 2024) and has shown no adverse effects in canines (Henriquez‐Hernandez et al. 2024).

The study began on 10 May, 2024, and ended on June 6, with a total of 10 doses administered on the following dates: May 10, 13, 16, 19, 22, 25, 28, 31, and June 3 and 6. This treatment protocol, consisting of 1 day of dosing followed by 2 days of rest, is commonly used in human medicine to manage anxiety through psychedelics microdosing (Fadiman 2011). Given the lack of previous veterinary studies, we adopted this protocol as a therapeutic approach. This conservative 2‐day dosing interval was chosen to prioritize animal safety.

1cp‐LSD was legally acquired through an online supplier (AlphaChain B.V., Utrecht, the Netherlands). According to the manufacturer, each pill contains 10 µg of 1cP‐LSD L‐tartrate, as determined by quantitative NMR spectroscopy. Each pellet is designed to be cut and dosed at 5 µg. The substance was dosed and prepared for administration in individual containers. It was agreed with the owner that the administration should be done, if possible, at the same time, with 9 AM being the chosen hour. Thus, the substance was administered by disguising it in a piece of ham and delivered orally to the animal at breakfast time.

The research team closely monitored the treatment's progress and maintained constant communication with the animal's owner throughout the study. No adverse effects were recorded.

### 2.4 Minimization of Observer Bias and Environmental Confounders

The lack of a placebo‐controlled, blinded design is a key limitation. To reduce observer bias and environmental confounders, several methodological precautions were implemented. Anxiety assessments were conducted using validated behavioural scales, ensuring structured and standardized evaluations. To maintain consistency, all observations took place in the dog's habitual environment, under controlled conditions, with the same owner, routine, and time of day for each assessment. Finally, to mitigate expectation bias, anxiety‐related behaviours were evaluated based on both subjective questionnaire responses and objective indicators, such as vocalization duration and destructive behaviours. These measurable parameters provided complementary evidence beyond owner perception.

## 3 Results

### 3.1 Owner's Attachment Evaluation

**FIGURE 1:**
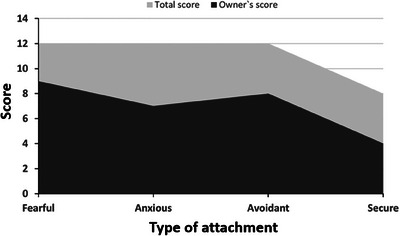
Area chart showing the different scores obtained by the owner of the animal in the various attachment subscales (dark grey). Maximum possible scores are included (light grey). The questionnaire is answered using a 4‐point Likert scale (Casullo and Fernández‐Liporace 2005): 1, Almost never; 2, Sometimes; 3, Often; and 4, Almost always. Three questions assess fearful attachment: (1) Even if I want to, it is hard for me to trust another person; (2) I think that affectionate relationships can hurt me; and (3) Committing to stable relationships scares me. Three questions assess anxious attachment: (1) I want to form affectionate relationships with someone, but I generally feel rejected; (2) I feel that others do not value me as much as I value them; and (3) I feel bad when I don't have true affectionate relationships. Three questions assess avoidant attachment: (1) I feel good when I avoid emotional commitments with other people; (2) I need to feel independent, without emotional commitments; and (3) I am uncomfortable depending emotionally on another person and having them depend on me. Two questions assess secure attachment: (1) I worry little about being alone, and (2) I worry little about feeling rejected.

The analysis of the owner's attachment questionnaire revealed a fearful/avoidant profile with a high anxious component, scoring 9/12, 8/12, and 7/12 for fearful, anxious, and avoidant attachment subscales, respectively (Figure 1). The lowest score was for secure attachment, with 4/8 points.

To the questions “I think emotional relationships can hurt me” and “I feel uncomfortable depending emotionally on someone,” the respondent answered “Almost always” (4 points). Conversely, to the question “I desire to emotionally connect with someone, but generally feel rejected,” the respondent answered “Almost never” (1 point). However, the respondent did say “Often feeling bad when not in true emotional relationships” and “Often feeling afraid to commit in emotional relationships.” This fearful‐avoidant profile with an anxious component indicates an insecure connection, characterized by a fear of intimacy and a simultaneous need for closeness.

### 3.2 Baseline Anxiety State of the Patient

**FIGURE 2:**
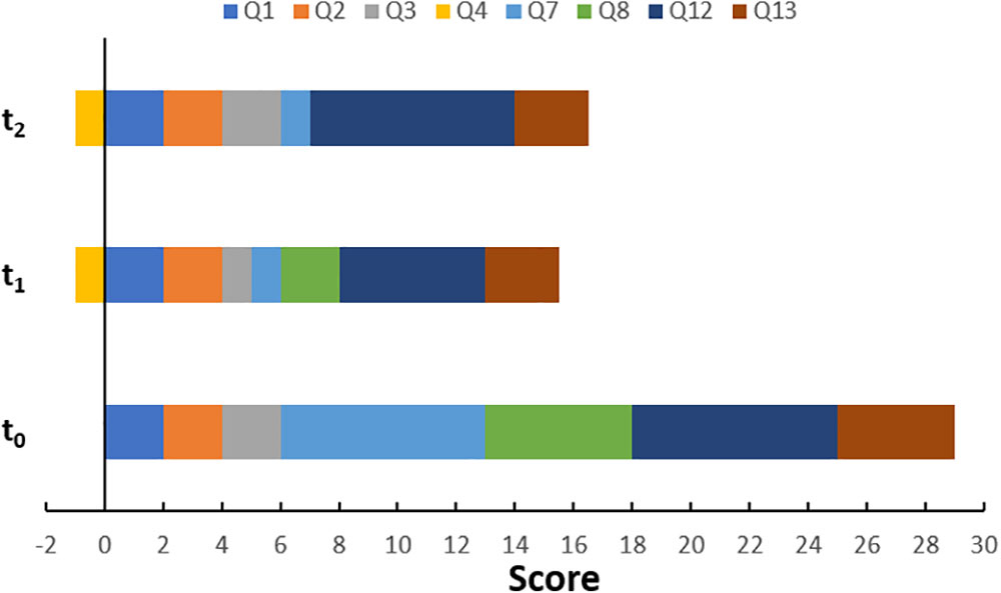
Stacked bar chart showing the scores obtained in the anxiety questionnaire before the start of treatment (*t*_0_), at the end of treatment (*t*_1_), and 1 month after the end of treatment (*t*_2_). Only the scored questions of the scale are shown.

The results of the patient's anxiety assessment are contained in Table 1. The dog's total score was 29 points, indicating severe separation anxiety (Figure 2). The dog exhibits notable behaviours when the owner is about to leave the house (Q1), including pacing, trembling, and whining, with whining being the most prominent. Similarly, once outside, the dog whines (Q2), which occurs more than once a day (Q12) and for more than 60 min (Q13). In addition to whining, barking is reported (Q14). The owner reported the dog's anxious attachment, mentioning that the dog follows her from room to room when at home (Q5). Additionally, it's important to note the dog's destructive behaviour, including breaking things (crates, its own bed) more than once a day (Q7), with this damage being extensive (Q8). On the other hand, the dog does not urinate/defecate or drool when the owner is away from home (Q9 and Q15, respectively).

**TABLE 1:** Anxiety assessment before the start of treatment (*t*_0_), at the end of treatment (*t*_1_), and 1 month after completion (*t*_2_). The scores assigned to each item were included.

	Scorea	T_0_	T_1_	T_2_
1. What does your dog do while you are getting ready to leave the house?				
Ignores you	0			
Watches you	0.5	x	x	x
Paces back and forth	2	x	x	
Whines or whimpers	2	**x**	**x**	**x**
Salivates	2			
Appears anxious or depressed	2	x	x	x
Trembles	2	x		
Other (panting/barking)	1	x		x
2. What does your dog do while you are leaving the house?				
No reaction	0			
Looks out the window	0.5			
Scratches at the door and/or window or crate	2			
Bites or scratches the door	2			
Vocalizes	2	x	x	x
3. What does your dog commonly do when you return home?				
Ignores you	0			
Greets you by licking or jumping on you (<1 min)	0.5		x	
Jumps on you less than a minute	1			
Vocalizes (barks/growls) less than a minute	1			
Follows you around the house less than a minute	1		**x**	
Greets you by licking or jumping on you (> 1 min)	2	x		x
Jumps on you for a minute or more	2			
Vocalizes for a minute or more	2			**x**
4. While you are at home, does your dog do any of the following behaviours?				
Excessively salivates	−1			
Urinates or defecates in the house	−1			
Destroys things	−1			
Excessively vocalizes	−1		x	x
None of the above	0	x		
5. What does your dog do most of the time while you are at home?^b^				
Ignores you	NS			
Stays in another room	NS		x	
Stays in other rooms and in the room where you are	NS		x	x
Stays in the room where you are	NS			
Follows you from room to room	NS	x		
Maintains physical contact with you	NS			
6. After reaching one year of age, has your dog destroyed anything while you were NOT at home?				
Yes	NS	x	x	x
No	NS			
7. How often has your dog destroyed any object in the past month?				
Less than once a month	1		x	x
1 to 2 times a month	1.5			
3 to 4 times a month	2			
5 to 7 times a month	2.5			
2 to 6 times a week	4			
Once a day	5			
More than once a day	7	x		
8. How severe were those destructive events?				
Small scratches or bites	1			
Small to intermediate scratches or bites	2		x	
Intermediate scratches and bites	3			
Intermediate to extensive scratches and bites	4			
Extensive scratches and bites	5	x		
9. After reaching one year of age, has your dog urinated or defecated while you were NOT at home?				
Yes	NS			
No	NS	x	x	x
11. After reaching one year of age, has your dog vocalized while you were NOT at home?				
Yes	NS	x	x	x
No	NS			
12. How often has your dog vocalized (whined, howled, or barked) in the past month?				
Less than once a month	1			
1 to 2 times a month	1.5			
3 to 4 times a month	2			
5 to 7 times a month	2.5			
2 to 6 times a week	4			
Once a day	5		x	
More than once a day	7	x		x
13. What is the approximate duration of the vocalizations while you are not at home?				
Less than 2 min	1			
2 to 5 min	1.5			
5 to 10 min	2			
10 to 20 min	2.5		x	x
20 to 30 min	3			
30 to 60 min	3.5			
More than 60 min	4	x		
14. What type of vocalization does your dog mainly make while you are not at home?^b^				
Whining	NS		x	x
Growling	NS			
Barking	NS	x	x	
Howling	NS		x	
Other	NS			
15. After reaching one year of age, does your dog excessively salivate while it has been alone at home?				
Yes	NS			
No	NS	x	x	x

*Note*: Q10 (How often have you had problems with urine/faeces in the past month?), Q16 (What is the extent of the salivation?) and Q17 (How often has your dog excessively salivated in the past month?) were omitted from the table as the patient neither urinates nor salivates when the owner is not home.

^a^In cases of multiple answers, the one with the highest value and/or the one most representative of the animal's behaviour was taken (X bold).

^b^It does not have a score as these are bonding disorders related to separation anxiety (SA), but they are not used in this scale.

Abbreviation: NS, not scored.

### 3.3 Treatment Progress

An audiovisual summary of the treatment can be found in Video S1, available at: https://www.youtube.com/watch?v=3qK0QGXDWyw. This is a 2.5 min video that includes images of the patient throughout the treatment month. Each video sequence is contextualized with accompanying commentary, and the specific date and time of each frame are clearly marked.

Session 1 was conducted on 10 May, 2024, and started at 9:30 AM, with the owner reporting nothing different from what was observed during the pilot test conducted a few months earlier (Henriquez‐Hernandez et al. 2024). During the second session, the owner mentioned that “something has been different.” Although the progression was similar, the animal had interacted considerably with its surrounding environment (Video S1, time interval (TI): 00:09–00:26). The owner had to travel on the day of the third administration (session 3), so she administered the treatment at 6:30 AM. A family member, known to the dog, stayed with her, and no significant actions were reported. By 10:15 AM, the dog was alert and “as usual,” having passed the relaxation phase that occurs between 1.5 and 2.5 h after ingestion (Video S1, TI: 00:27–00:35) (Henriquez‐Hernandez et al. 2024).

Session 4 started at 9:00 AM, May 19. At 11:00 AM, the owner noticed unusual behaviour: the dog called from upstairs, not coming down (Video S1, TI: 00:36 – 01:02). The owner mentioned the dog typically whined when she went down, not called. She complied, going up with the dog. Immediately afterwards, the patient went to rest. By 12:00 PM, the dog explored (Video S1, TI: 01:03–01:39) and then lay down. At 1:00 PM, she remained relaxed. Shortly after, the owner said, “she's back”. In session 5 (May 22), everything proceeded normally. At noon, 3 h after administration, the owner left the house, and the dog barked at the door, though with less insistence. In session 6 (May 25), the trial was conducted in the presence of another person (the owner's partner), as the owner would be away for a few days. This person is familiar with the dog, and no significant events were reported.

Two photos of a broken crate and cushion were reported (Video S1, TI: 01:40–01:50), with the damage occurring during the owner's absence. She questions the treatment and wonders if it's “counterproductive.” The owner's father, who also suffers from anxiety, is staying at the house for several days. The owner is asked about her anxiety condition. She reports a peak in personal anxiety due to a job interview on May 16, saying, “Today my chest feels tight.” She acknowledges going through a difficult and tumultuous stage in her life, but still trusts the research team. The dog remained calm during session 7 on May 28.

The owner was away from May 30 to June 2. Her father, who also suffers from anxiety, stayed with the dog and administered session number 8 on May 31. He reported that the previous day, he had taken the other dog for a walk, and the patient did not stop barking. On the day of administration, the dog retired to rest 2 h after ingestion. She appeared affectionate yet relaxed. He left her alone at times, and she did not follow him but remained alert, “locating” the person who was with her.

Due to a personal issue, the administration was delayed until 3:20 PM, June 3 (session 9). By 4:50 PM, the dog was playful and alert (Video S1, TI: 01:51–02:10). At 5:55 PM, the owner reported: “Not stopping today, she seems tired, panting, and restless. She wandered around but without whining or crying.” At 6:00 PM, finally, she settled in her spot to rest (Video S1, TI: 02:11 – 02:15). At 6:15 PM, she retired to her room. By 7:00 PM, she was active again.

The last session took place on June 6. The substance was administered at 9 AM. The patient remained calm until 10:30. She sought affection and returned to her room. When the other dog she lives with barked, she sought comfort. She stayed in the room until noon, at which point the owner and her father went out shopping. The patient left her resting place and barked at the door. She was taken for a walk at 7:20 PM (Video S1, TI: 02:16–02:22), with the following observation: compared with her usual behaviour, we agree that she is calmer than other times, but still, she does not stop.

### 3.4 Posttreatment Anxiety Assessment

**FIGURE 3:**
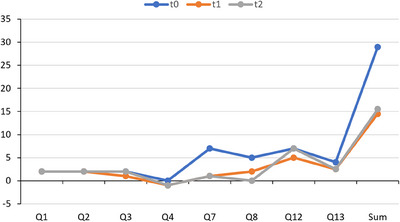
Changes in the scores obtained in the anxiety questionnaire before the start of treatment (*t*_0_ in blue), at the end of treatment (*t*_1_ in orange), and 1 month after the end of treatment (*t*_2_ in grey). Only the scored questions of the scale are shown. The “Sum” indicates the total value across all scored items at each time point.

The results of the patient's post‐treatment anxiety assessment are contained in Table 1. The dog's total score was 14 points, indicating moderate anxiety (Figure 2). The main differences were found in the following questions (Figure 3). Q1 (What does your dog do while you are getting ready to leave the house?): No tremors were noted. Q3 (What does your dog commonly do when you return home?): From 2 points before the treatment (greets by licking or jumping on you for more than a minute), it went to 1 (follows you around the house for less than a minute).

Q4 (While you are at home, does your dog do any of the following behaviours?): From scoring 0 (None of the above), it moved to ‐1 (overly vocalizes). Q5 (What does your dog do most of the time while you are at home?): Before treatment, it was reported “follows you from room to room”. This behaviour was not observed after treatment (*t*_1_). It has been replaced by “remains in other rooms and within the room where you are”, with no score assigned to this question.

There was a significant change recorded regarding Q7 (How often has your dog destroyed any object in the past month?): it went from more than once a day to less than once a month. Referring to the intensity of these destructive events (Q8), there was a shift from “extensive bites and scratches” to “small or moderate scratches and bites”, scored with 5 and 2 points, respectively. Q12 (How often has your dog vocalized in the past month?): It changed from more than once a day to once a day. Finally, referring to Q13 (What is the approximate duration of the vocalizations while you are not at home?), it changed from more than 60 min to 10–20 min, for *t*_0_ and *t*_1_, respectively (Figure 3).

On July 7th, 1 month after the end of treatment, the patient's final anxiety assessment was conducted. The results were similar to the previous test, with a total score of 15.5 points, equivalent to the border between moderate and marked anxiety (Figure 2). The most significant changes were observed in questions 3 (What does your dog commonly do when you return home?) and 12 (How often has your dog vocalized in the last month?) (Figure 3). In comparison, a notable increase in the duration and frequency of the anxious response was observed. This was characterized by vocalization lasting over 1 min and occurring multiple times per day (Table 1). Regarding question 8 (How severe have these destructive events been?), no such events were recorded. While the owners clarify that they try not to leave anything within the dog's reach, during the treatment, the only episode that occurred was much milder than usual (Figure 3). No changes were observed in the remaining questions compared with the anxiety assessment conducted at the end of treatment (*t*_1_ vs. *t*_2_, Figure 3).

### 3.5 Reflections From the Owner and Researchers

The owner was asked to provide a final reflection on the experience. After completing the treatment (t_1_), she mentioned observing the dog being generally less anxious. One month later (t_2_), she reported that the dog seems calmer. These findings are consistent with the data presented in Table 1, which reveals alterations in vocalization duration (questions 12 and 13), vocalization type (question 14), and the dog's behaviour in the owner's presence (question 5). The experience significantly increased her awareness of how her own emotional state affects her dog. A sense of guilt regarding this reflection was evident in the patient's owner. The observed results may be biased because the owner knew what substance the animal was taking, highlighting the importance of contrasting these results with a blind study where owners are unaware of whether their pets are receiving a placebo or 1cp‐LSD. The patient's anxiety peaks (sessions 7 and 9) coincided with those reported by the owner, which reinforces the thesis establishing a relationship between the dog's character/behaviour and that of the owner. It should be noted that administering 1cp‐LSD in the afternoon had less anxiolytic effect, which opens significant avenues for further research in this regard.

## 4 Discussion

The success of treating certain human mental health conditions with psychedelic substances lies, among other factors, in the intentionality of the intake and the ability to psychologically work with the individual before, during, and especially after the psychedelic experience (Borkel et al. 2024). This approach cannot be carried out with any other species. However, psychedelic substances have the ability to modulate animal behaviour (Trulson and Howell 1984). It has been hypothesized that hallucinogenic plants alter the perception of hunting dogs by decreasing the reception of external stimuli and increasing sensory perception, especially their sense of smell. This theory explains the ancient use of various plant varieties by South American tribes to enhance the effectiveness of hunting dogs (Bennett and Alarcon 2015, Jernigan 2009). The study of the effects of psychedelics on animal intelligence was epitomized by John C. Lilly, whose experiments with dolphins formed the basis for considering them especially intelligent (Peters 2020). However, with few exceptions, most experiments conducted on animals are aimed at understanding the physiology, pharmacology, and toxicology of psychedelic substances, without paying attention to the potential benefits these therapies might have on animal welfare. In that sense, the administration of psychedelics to animals raises significant ethical considerations that warrant careful examination. While this study aimed to explore the potential therapeutic benefits of these substances, it is crucial to acknowledge the potential impact on animal welfare. The long‐term effects of repeated psychedelic administration in animals are unknown, and further research is needed to assess potential adverse effects. The use of psychedelics in animals raises questions about informed consent and the ability of animals to comprehend the nature and purpose of the study.

Anxiety is prevalent in dogs, affecting an estimated one‐fifth of the canine population (Salonen et al. 2020). While a variety of pharmacological treatments exist, their efficacy is not guaranteed (Sherman and Mills 2008, Dantas and Ogata 2024). The associated distress experienced by owners can lead to negative outcomes for the dog, including abandonment or rehoming (Crespo‐Garay 2021). This situation has expanded the spectrum of potential pharmacological treatments for this condition. Among them, therapies using natural medicines have recently begun to be used, having patented some active ingredients (Liu et al. 2017). This is the case of cannabidiol (CBD), whose use for reducing canine anxiety has become popular (Hunt et al. 2023) but shows controversial results (Yu and Rupasinghe 2021). Similar situations have occurred in human medicine: high rates of mental disorders (primarily anxiety and depression), limited success rates, and adverse effects in terms of addiction and tolerance (Lader 1994) have underscored the need for alternative therapies. Unlike in humans, in whom psychological therapies improve the prognosis of such disorders (Aderka et al. 2012), behavioural interventions performed by ethologists are not cognitively equivalent. The therapeutic potential of psychedelic substances in the field of psychiatry has been known for decades. The implications for veterinary pharmacology of our findings highlight the potential of psychedelics as novel therapeutic agents for managing anxiety‐related disorders in companion animals. Given the limited pharmacological options currently available, the observed efficacy of low‐dose 1cp‐LSD suggests a new avenue for therapeutic intervention that warrants further investigation. The mechanism of action of psychedelics, particularly their modulation of serotonergic pathways, aligns with existing anxiolytic strategies but may offer advantages in terms of efficacy and tolerability. However, the potential role of neuroinflammation or neurological alterations due, for example, to *Toxoplasma gondii* infection, raises important questions about whether neurobiological factors beyond serotonin dysfunction—such as immune or inflammatory pathways—could influence anxiety manifestations in dogs (Dini et al. 2025).

Anxiety is a complex condition determined by both genetic and environmental components. The parallel between the pathophysiology of human and canine anxiety justifies the use of psychedelic substances for controlling anxiety in dogs (Vermeire et al. 2009). In this context, lysergic acid diethylamide (LSD) has proven to be useful (Lowe et al. 2022). In the present study, we opted for a 1‐month treatment following the Fadiman protocol (Fadiman 2011), microdosing a legal analogue of LSD: 1cp‐LSD (Brandt et al. 2020). This approach allows us to (i) observe behavioural changes over a longer period and (ii) avoid exposing the animal to a high dose of psychedelics, which could induce an undesired and incomprehensible experience along with its associated risks (Aday et al. 2021). In the present study, a total of 5 µg was administered orally to the patient, equivalent to 0.38 µg/kg of body weight. For an 80 kg person, this represents a dose of 21.4 µg, considered in the upper limit of a typical microdosing range (5–25 µg) (Liechti and Holze 2022). The dog experienced psychedelic effects without adverse reactions (restlessness, vocalization, and confusion), remaining conscious and responsive. This suggests an appropriate dosage, well within the microdosing range, as full psychedelic effects typically require 100–500 µg (Liechti and Holze 2022).

Our results show a reduction in anxiety, decreasing from the initial 29 points to 14.5 at the end of the treatment. Although this observation supports the initial hypothesis, it requires further clarification. In our opinion, two elements should be highlighted. First, since the owner knew what was being administered to her pet, there is a possibility of observational bias. The owner answered the questions and might have unintentionally inferred an improvement. Similarly to how psychedelic research can be influenced by the experiences of the researchers themselves, a similar bias could occur between the owner and their pet (Sanders and Zijlmans 2021, Nielson and Guss 2018). However, it should be noted that the scores associated with each question were unknown to the owner, and she did not have access to the interpretation of the questionnaires. She was not informed of anything until after completing the three anxiety evaluations to avoid any form of conditioning. The potential placebo effect, both in terms of owner perception and the dog's response to increased attention and routine changes, has to be highlighted. This aspect may be crucial in interpreting the results, as previous studies have already demonstrated that this phenomenon exists in the context of veterinary clinical research and may obscure the robustness of the observed findings (van Haselen and Jutte 2013). Second, the coexistence of human and animal anxiety is a crucial factor to consider when evaluating results. The study design does not allow for causal inferences regarding the relationship between the dog's behaviour and the owner's emotional state. Our findings suggest an association between these variables, but establishing causality would require longitudinal studies with controlled experimental conditions. In that line, previous studies have demonstrated that animal welfare and human well‐being are closely related (Amiot and Bastian 2023). Therefore, future research should include parallel monitoring of the owners’ emotional state alongside that of their dogs. Owners may project improvement in their own symptoms—if applicable—onto their pet. Conversely, temporary worsening of anxiety in the owner can significantly influence the anxiety experienced by the dog (Xestal 2022, Pereira et al. 2021, O'farrell 1987). This association was observed in the current study. According to reports from the owner, sessions with less effect (sessions 6 and 9), as well as the only instance of destructive behaviour by the patient throughout the treatment (between sessions 6 and 7), occurred when the owner reported higher anxiety (related to a job interview), when another person with anxiety (her father) was introduced into the dog's environment, or when the animal's routines were altered (absence due to travel). Session 9 also occurred in the afternoon. While the specific factor influencing the dog's experience cannot be determined, this event is noteworthy.

The anxiolytic effect was sustained for 1 month after the treatment's completion. The sustained long‐term effects of psychedelic microdosing treatments are still under investigation. In humans, these effects have been observed to persist for up to 4 months after treatment (Fadiman and Korb 2019), although there is some debate about this (Polito and Stevenson 2019). Therefore, although the present results suggested that the anxiolytic effect may persist 1 month after treatment completion, this hypothesis must be demonstrated in future studies. This outcome is intriguing because, while it is not feasible to assess the intentionality of medication intake in animals, a crucial factor in the success of such therapies in humans (Borkel et al. 2024), it suggests that behavioural modifications may have a biological rather than primarily psychological basis. This opens a discussion regarding the level of animal consciousness, particularly in the canine species (Birch et al. 2020, Gregory 2003), which additionally experience different levels of consciousness according to the breed they belong to (Accorsi et al. 2017). While the neurobiological mechanisms underlying the effects of psychedelics are largely conserved across mammals (Bennett and Alarcon 2015, Liu et al. 2017), significant differences in cognition and consciousness between humans and canines must be acknowledged. The subjective experience of altered states in animals remains unknown, and behavioural assessments are inherently limited in capturing potential perceptual or emotional shifts. Given these uncertainties, caution is warranted when extrapolating findings from human studies to veterinary applications. Furthermore, ethical concerns arise when administering substances with limited prior pharmacological data in a given species. However, off‐label drug use is a common necessity in veterinary medicine, particularly in conditions with suboptimal treatment options. This study was designed with safety as the primary consideration, employing a conservative dosing regimen and close monitoring to minimize risk. A thorough understanding of the pharmacokinetics and long‐term effects of psychedelics in canines is essential to ensure both efficacy and ethical responsibility in their potential therapeutic use.

Taken together, we propose future research in several directions. First, conducting a blinded efficacy trial with an appropriate number of dogs, ensuring that owners are unaware of whether their pet is in the treatment or placebo group. Second, focus should be placed on assessing the owners’ mood and examining their potential influence on the dogs. Finally, social media can play a crucial role in addressing misinformation and public skepticism surrounding psychedelic therapies. Digital platforms such as Instagram have proven effective in enhancing scientific communication, particularly in veterinary contexts. Previous educational initiatives have demonstrated high levels of engagement and knowledge retention among veterinary students and professionals, confirming the value of these platforms in translating complex concepts into accessible content (Lamanna et al. 2025). In this regard, future dissemination efforts for innovative therapeutic strategies, such as microdosing psychedelics in veterinary medicine, should consider targeted social media campaigns to foster public understanding, professional awareness, and informed discussion.

## 5 Strengths and Limitations

This study has several limitations that must be considered when interpreting the results. First, the lack of pharmacological studies of the substance in canines means that both the dosage and treatment protocol may not be optimal. The drug's half‐life and effective dose 50 are unknown, making it difficult to determine the appropriate dosage and treatment regimen. To address this limitation, we emulated the human dosage and employed a conservative treatment protocol to prioritize patient safety. Specifically, a microdose comparable to human microdosing was administered once every 3 days. Second, as this is a single‐case study, there is no control (placebo) group and treatment group, which does not allow for definitive conclusions about the substance's actual role. Due to the limited existing research in this area, we opted for a stepwise approach before undertaking a larger clinical trial. This involved an initial single‐day experiment (Henriquez‐Hernandez et al. 2024) followed by a comprehensive month‐long study, the results of which are presented herein. Indeed, following this report, a clinical trial is the next step. Finally, as this study is based on a single case without repeated measurements, the use of inferential statistical tests is not applicable. The magnitude of change in the anxiety score is purely qualitative, and the results may be anecdotal and spurious. Moreover, the owner's emotional state was not assessed throughout the experimental period. While previous research suggests a strong relationship between human and canine emotional states, our study design did not allow for a quantitative assessment of this interaction. Future studies should incorporate longitudinal monitoring of both the dog's anxiety levels and the owner's psychological state to better understand this dynamic relationship. The potential placebo effect, both in terms of owner perception and the dog's response to increased attention and routine changes, must be taken into account in future research.

Conversely, the present study offers several notable strengths. Despite being a single‐subject case study, the replication of human medical findings, including the treatment regimen and dosage, attests to the robustness of our approach. Additionally, the employment of validated scales for assessing both canine anxiety and owner attachment style ensures a rigorous and objective methodology. Moreover, the utilization of legal analogues of the substance facilitates accessibility and enables future replication or in‐depth exploration of the findings. Notably, this approach to treating canine anxiety establishes a foundation upon which further research in this domain can be built.

## 6 Conclusion

The administration of 5 µg of 1cp‐LSD once every 3 days over a 30‐day period was associated with a reduction in severe anxiety to a moderate level in a female dog, with the effect persisting for 1 month posttreatment. Given the exploratory nature of this single‐case study, these findings should be interpreted with caution. The role of owner‐related psychological factors as well as contextual variables (e.g., presence of other people, timing of administration) warrants further investigation. Placebo‐controlled trials are necessary to substantiate these preliminary observations and assess the therapeutic potential of 1cp‐LSD for canine anxiety.

## Author Contributions

Conceptualization: Luis Alberto Henríquez‐Hernández. Data curation: Elisa Hernández‐Álvarez and Ignacio García‐Serrano. Formal analysis: Elisa Hernández‐Álvarez and Luis Alberto Henríquez‐Hernández. Investigation: Elisa Hernández‐Álvarez, Lucas F. Borkel, Jaime Rojas‐Hernández, Domingo J. Quintana‐Hernández, Tobías Fernández‐Borkel, and Luis Alberto Henríquez‐Hernández. Methodology: Ignacio García‐Serrano, Manuel Zumbado, and Luis Alberto Henríquez‐Hernández. Project administration: Manuel Zumbado and Luis Alberto Henríquez‐Hernández. Resources: Lucas F. Borkel, Jaime Rojas‐Hernández, and Luis Alberto Henríquez‐Hernández. Supervision: Luis Alberto Henríquez‐Hernández. Visualization: Elisa Hernández‐Álvarez and Luis Alberto Henríquez‐Hernández. Writing—original draft: Luis Alberto Henríquez‐Hernández. Writing—review & editing: Elisa Hernández‐Álvarez, Lucas F. Borkel, Jaime Rojas‐Hernández, Domingo J. Quintana‐Hernández, Ignacio García‐Serrano, Tobías Fernández‐Borkel, and Manuel Zumbado.

## Institutional Review Board Statement

The study was approved by the Animal Experimentation Ethics Committee of the University of Las Palmas de Gran Canaria (ref no. OEBA_ULPGC 02/2024).

## Informed Consent Statement

The animal's owner signed the necessary informed consent before the trial began.

## Conflicts of Interest

The authors declare no conflicts of interest.

## Supporting information




**Table S1**. Spanish translation of the validated original canine anxiety scale. The scores assigned to each item are indicated in parentheses.

## Data Availability

Data available on request from the authors. The data that support the findings of this study are available from the corresponding author upon reasonable request.
