# Supplementary material for: Evaluating the Potential of Microdosing 1cp‐LSD for the Treatment of Canine Anxiety: A One‐Month Case Study
Source: Vet Med Sci. 2025 Jul 10;11(4):e70486. doi: 10.1002/vms3.70486 (PMC12244264; doi:10.1002/vms3.70486)
Supplement: Supplementary file 1 — Table S1. Spanish translation of the validated original canine anxiety scale. The scores assigned to each item are indicated in parentheses. [file VMS3-11-e70486-s001.docx]

**Table S1.** Spanish translation of the validated original canine anxiety scale. The scores assigned to each item are indicated in parentheses.

| **1. ¿Qué hace su perro mientras usted se prepara para salir de casa?** | |
| --- | --- |
| □ Lo ignora (0) |  |
| □ Lo observa (0.5) |  |
| □ Camina de un lado para otro (2) |  |
| □ Se queja o emite gemidos (2) |  |
| □ Saliva (2) |  |
| □ Se ve ansioso o deprimido (2) |  |
| □ Tiembla (2) |  |
| □ Otras (1) |  |
| **2. ¿Qué hace el perro mientras sale de su casa?** | |
| □ No presenta reacción (0) |  |
| □ Mira por la ventana (0.5) |  |
| □ Rasguña la(s) puerta(s) y/o ventana(s) o su jaula de transporte (2) |  |
| □ Muerde o araña la puerta (2) |  |
| □ Vocaliza (gime, ladra o aúlla) (2) |  |
| **3. ¿Qué hace comúnmente su perro cuando regresas a casa?** | |
| □ Lo ignora (0) |  |
| □ Lo saluda lamiéndole o saltando sobre usted – menos de un minuto (0,5) |  |
| □ Salta sobre usted menos de un minuto (1) |  |
| □ Vocaliza (ladra/gruñe) menos de un minuto (1) |  |
| □ Lo sigue por la casa menos de un minuto (1) |  |
| □ Lo saluda lamiéndole o saltando sobre usted – más de un minuto (2) |  |
| □ Salta sobre usted por un minuto o más (2) |  |
| □ Vocaliza por un minuto o más (2) |  |
| **4. Mientras usted se encuentra en la casa, ¿su perro hace alguna de las siguientes conductas?** | |
| □ Saliva excesivamente (-1) |  |
| □ Orina o defeca en casa (-1) |  |
| □ Destruye cosas (-1) |  |
| □ Vocaliza excesivamente (-1) |  |
| □ Ninguna de las anteriores (0) |  |
| **5. ¿Qué hace su perro la mayoría del tiempo mientras están en casa?*** | |
| □ Lo ignora |  |
| □ Permanece en otra habitación |  |
| □ Permanece en otras habitaciones y dentro de la habitación donde usted se encuentra |  |
| □ Permanece en la habitación donde usted se encuentra |  |
| □ Lo sigue de habitación en habitación |  |
| □ Mantiene un contacto físico con usted |  |
| **6. Tras haber cumplido un año de edad, ¿su perro ha destruido algo mientras usted NO ESTÁ en la casa?** | |
| □ Sí |  |
| □ No |  |
| **7. ¿Con que frecuencia ha destruido algún objeto este último mes?** | |
| □ Menos de una vez al mes (1) |  |
| □ 1 a 2 veces al mes (1,5) |  |
| □ 3 a 4 veces al mes (2) |  |
| □ 5 a 7 veces al mes (2.5) |  |
| □ 2 a 6 veces a la semana (4) |  |
| □ 1 vez al día (5) |  |
| □ Más de una vez al día (7) |  |
| **8. ¿Cómo de graves o severos han sido esos eventos de destructividad?** | |
| □ Rasguños a mordeduras pequeños (1) |  |
| □ Rasguños a mordeduras entre pequeños e intermedios (2) |  |
| □ Rasguños y mordeduras Intermedio (3) |  |
| □ Mordeduras y rasguños entre intermedios y extensos (4) |  |
| □ Mordeduras y rasguños extensos (5) |  |
| **9. Tras haber cumplido un año de edad, ¿se ha orinado o defecado su perro mientras usted NO ESTÁ en la casa?** | |
| □ Sí |  |
| □ No |  |
| **10. ¿Con qué frecuencia ha tenido problemas con la orina/heces este último mes?** | |
| □ Menos de una vez al mes (1) |  |
| □ 1 a 2 veces al mes (1,5) |  |
| □ 3 a 4 veces al mes (2) |  |
| □ 5 a 7 veces al mes (2,5) |  |
| □ 2 a 6 veces a la semana (4) |  |
| □ 1 vez al día (5) |  |
| □ Más de una vez al día (7) |  |
| **11. Tras haber cumplido un año de edad, ¿ha vocalizado su perro mientras usted NO ESTÁS en la casa?** | |
| □ Sí |  |
| □ No |  |
| **12. ¿Con que frecuencia ha vocalizado (gemido, aullido o ladrido) este último mes?** | |
| □ Menos de una vez al mes (1) |  |
| □ 1 a 2 veces al mes (1,5) |  |
| □ 3 a 4 veces al mes (2) |  |
| □ 5 a 7 veces al mes (2,5) |  |
| □ 2 a 6 veces a la semana (4) |  |
| □ 1 vez al día (5) |  |
| □ Más de una vez al día (7) |  |
| **13. ¿Cuál es la duración aproximada de las vocalizaciones mientras usted no está?** | |
| □ Menos de dos minutos (1) |  |
| □ 2 a 5 minutos (1,5) |  |
| □ 5 a 10 minutos (2) |  |
| □ 10 a 20 minutos (2,5) |  |
| □ 20 a 30 minutos (3) |  |
| □ 30 a 60 minutos (3,5) |  |
| □ Más de 60 minutos (4) |  |
| **14. ¿Qué tipo de vocalización realiza principalmente mientras usted no está en casa?** | |
| □ Gemido |  |
| □ Gruñido |  |
| □ Ladrido |  |
| □ Aullido |  |
| □ Otro (diga cuál): |  |
| **15. Tras haber cumplido un año de edad, ¿saliva su perro excesivamente mientras ha estado solo en el hogar?** | |
| □ Sí |  |
| □ No |  |
| **16. ¿Cuál es el alcance de la salivación?** | |
| □ Húmedo alrededor de la boca (1) |  |
| □ Mojado alrededor de la boca (2) |  |
| □ Húmedo alrededor de la boca y patas delanteras (3) |  |
| □ Mojado alrededor de la boca y patas delanteras (4) |  |
| **17. ¿Con qué frecuencia ha tenido salivación excesiva este último mes?** | |
| □ Menos de una vez al mes (1) |  |
| □ 1 a 2 veces al mes (1,5) |  |
| □ 3 a 4 veces al mes (2) |  |
| □ 5 a 7 veces al mes (2.5) |  |
| □ 2 a 6 veces a la semana (4) |  |
| □ 1 vez al día (5) |  |
| □ Más de una vez al día (7) |  |

*Esta pregunta es para evaluar el hiper-apego o apego ansioso del perro. No tiene puntuación ya que son trastornos del vínculo relacionados con la ansiedad por separación (APS) pero que no se usan en esta escala.
